# 16S rRNA gene amplicon sequence data from sunflower endosphere bacterial community

**DOI:** 10.1016/j.dib.2021.107636

**Published:** 2021-11-26

**Authors:** Olubukola Oluranti Babalola, Bartholomew Saanu Adeleke, Ayansina Segun Ayangbenro

**Affiliations:** Food Security and Safety Niche Area, Faculty of Natural and Agricultural Sciences, North-West University, Private Bag X2046, Mmabatho 2735, South Africa

**Keywords:** DNA extraction, Endophytes, *Helianthus annus*, Illumina sequencing, South Africa

## Abstract

Insights into plant endosphere bacterial diversity and exploration of their bioincentives in the formulation of biofertilizers promise to avert ecological disturbances. Here, we presented the sequence dataset of the endophytic bacterial community from the roots and stems of sunflower obtained from farmlands in Itsoseng and Lichtenburg, North West Province of South Africa, using 16S rRNA gene amplicon sequencing. The climatic conditions of this region are characterized by an annual rainfall of about 600 mm and a temperature range of 3 to 21°C during winter and 22 to 34°C during summer. The genomic DNA was obtained from 1 g of each macerated sample using commercial DNA kits (DNeasy® Plant Mini kit, Qiagen, USA). The DNA was amplified through polymerase chain reaction at the V4 region using the specific forward and reverse primers. Amplicon sequencing was performed on the Miseq Illumina platform. Sequence read processing was performed using QIIME 1 16S-based pipeline implemented on Nephele microbial bioinformatics platform using default parameters. The sequence has been deposited in the Sequence Read Archive (SRA) of the National Center for Biotechnology Information (NCBI) with assigned Bioproject accession numbers. The data reveals the endophytic bacterial community structure and functions in sunflower cultivated in organic and nonorganic soils at growing and flowering stages.

**Specifications Table**

.

**Table:** 

Subject	Microbiology
Specific subject area	Bacteriology, Next-Generation Sequencing (NGS)
Type of data	Amplicon data (Table and Figure)
How data were acquired	Amplicon metagenome sequencing was performed with Illumina MiSeq platform, and OTU clustering analysis was conducted using the QIIME 1.
Data format	Raw data (fastq)
Parameters for data collection	The roots and stem of the sunflower were collected, and bacterial diversity and functions were analyzed from the extracted DNA using commercial DNA kits (DNeasy® Plant Mini kit, Qiagen, USA).
Description of data collection	Sequence read processing was performed using QIIME 1 16S-based pipeline implemented on the Nephele platform. The read preprocessing steps included pair joining using default parameters, removal of reads with an average Phred score of ≤ 20, removal of chimeras using VSEARCH, and clustering, using the Open Reference method and SILVA 99. Taxonomy assignments were performed against SILVA, with a default sequence similarity threshold of 0.99.
Data source location	Sunflower samples were collected from Itsoseng (S26°3′20.106′′E25°56′24.234) and Lichtenburg (S26°4′31.266′′ E25°58′44.442), North West Province, South Africa.
Data accessibility	The sequences have been deposited in the SRA of the NCBI with Bioproject assigned accession numbers, PRJNA673781 and PRJNA673791. The datasets generated for the OTU taxonomic assignment, OTU abundance, and predicted functions were implemented on Nephele microbial bioinformatics platform (version 1.8) (https://nephele.niaid.nih.gov/).

## Value of the Data

• Harnessing sunflower-associated bacteria endophytes with multifunctional attributes is promising in developing eco-friendly agriculture sustainably, although less explored.
• The data information provides the endophytic bacterial community of the sunflower at the growing and flowering stages in North West Province, South Africa, under different agricultural systems.
• This data can be employed to assess the roles of diverse bacterial species in maintaining endosphere competence for improved sunflower production.

## 1 Data Description

**Fig. 1:**
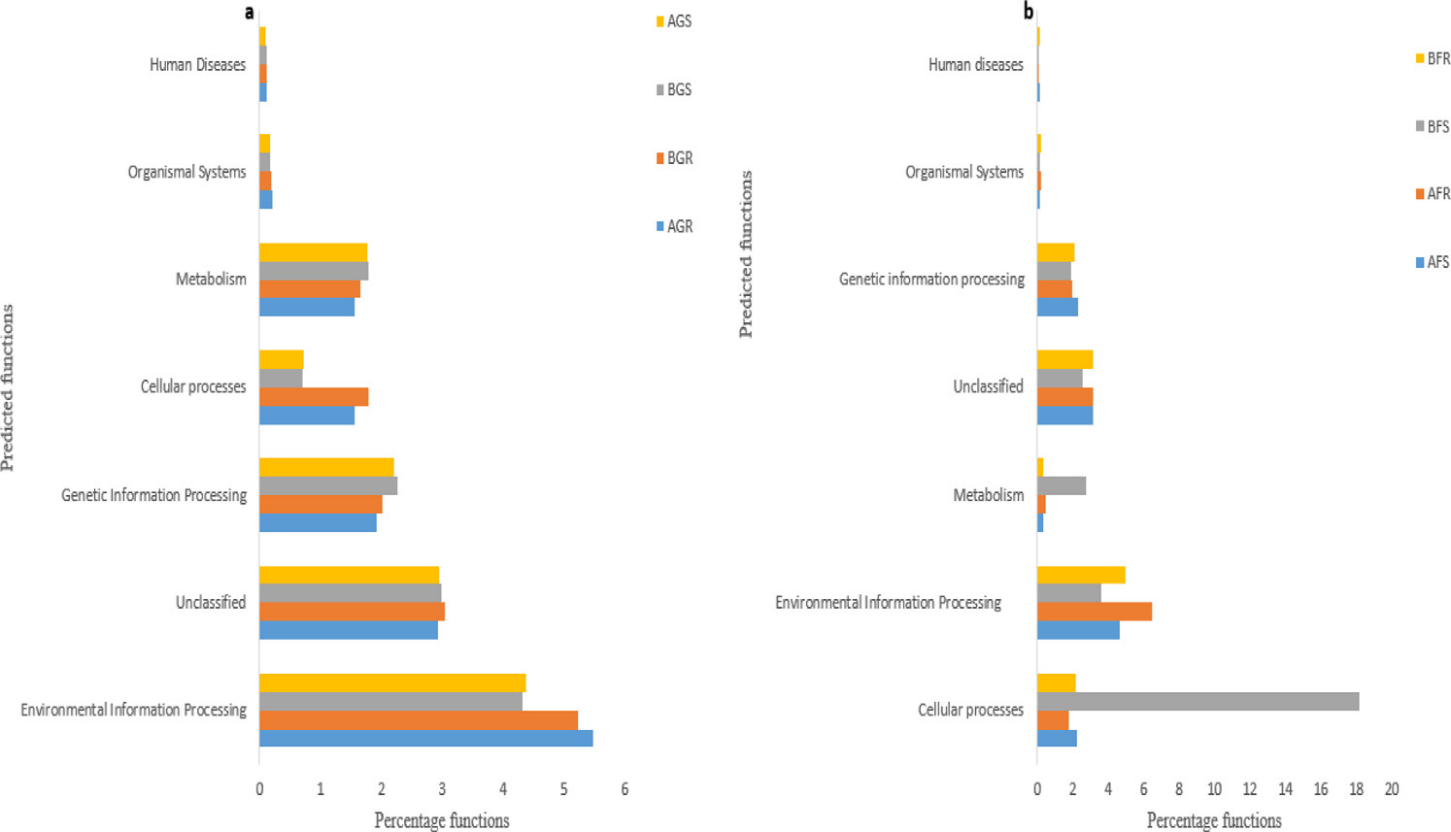
Predicted amplicon metagenome functions of bacterial endophytes in growing sunflower (a) and flowering sunflower (b). Key: Growing (AGR –root samples from Lichtenburg, BGR−root samples from Itsoseng, AGS−stem samples from Lichtenburg, BGS−stem samples from Itsoseng); Flowering (AFR –root samples from Lichtenburg, BFR−root samples from Itsoseng, AFS−stem samples from Lichtenburg, BFS−stem samples from Itsoseng).

The dataset described here comprises amplicon data information of bacterial community associated with the root and stem of sunflower from North West Province of South Africa. The detailed sequence datasets from NCBI can be accessed under Bioproject accession numbers PRJNA673781 and PRJNA673791. The 16S rRNA gene amplicon sequence reads information on bacterial diversity estimate in stem and root of sunflower are shown in Table 1 and Table S1, while the predicted bacteria functions are presented in Fig. 1 and Table S2, respectively. The downstream 16S rRNA gene amplicon analysis of sequence reads and basic information on diversity estimates using Nephele is represented in Table 1. The total read count obtained from growing sunflower was 237,439, with a median count of 57,259 per sample summary. The minimum and maximum counts per sample were 48,763 and 74,158, respectively. Also, in flowering sunflower, a total read count of 331,510 with 81,508 median counts per sample was recorded, while a read count of 60,480 and 108,015 was recorded minimum and maximum per sample. The OTU taxonomic assignment and OTU abundance of the sunflower at the growing and flowering stages are presented in Table S3a, S3b, S4a, S4b, S5a, and S5b. At level 1 selection, endophytic bacterial across the sites revealed varied functional attributes, which include metabolism, organismal systems, unclassified, cellular processes, environmental and genetic information processing (Fig. 1 and Table S2).

**Table 1:** 16S rRNA gene amplicon sequence reads information on diversity estimate in stem and root of sunflower plants.

Sample	NOS	NOS after QC	Barcode sequence	NCBI Bioproject accession number
**Growing**				https://www.ncbi.nlm.nih.gov/bioproject/PRJNA673791
AGR	74,158	2,497	AGR1 (AACAACCG)	
			AGR2 (AACAACGG)	
AGS	58,131	1,507	AGS1 (AACAAGAC)	
			AGS2 (AACAAGCT)	
BGR	48,763	1,566	BGR3 (CCATCGAA)	
			BGR4 (CCATGCTT)	
BGS	56,387	1,744	BGS3 (CCATTACC)	
			BGS4 (CCGCAATA)	
**Flowering**				https://www.ncbi.nlm.nih.gov/bioproject/PRJNA673781
AFR	73,286	2,519	AFR1 (CCGCTTAT)	
			AFR4 (CCGGAATT)	
AFS	60,480	1,833	AFS1 (CCGGTAAT)	
			AFS4 (CCGGTTAA)	
BFR	89,729	2,743	BFR2 (CCTAATCC)	
			BFR4 (CCTACGTT)	
BFS	108,015	3,488	BFS2 (CCTACCAT)	
			BFS4 (CCTAGCAA)	

**Key: Growing** (AGR –root samples from Lichtenburg, BGR−root samples from Itsoseng, AGS−stem samples from Lichtenburg, BGS−stem samples from Itsoseng); **Flowering** (AFR–root samples from Lichtenburg, BFR−root samples from Itsoseng, AFS−stem samples from Lichtenburg, BFS−stem samples from Itsoseng), NOS – number of sequences, QC – quality control.

## 2 Experimental Design, Materials and Methods

### 2.1 Sampling design and collection

Samples were carefully dug at a depth of 20 cm and uprooted from two sites (Itsoseng and Lichtenburg) from sunflower at growing and flowering stages. Plant samples at 4 weeks (growing) and 12 weeks (flowering) were gently uprooted from each site in triplicates and transported to the laboratory on icepacks. Collection of sunflower roots and stems was performed to determine the endophytic bacterial community structure and functions. Before surface-sterilization, plant roots and stems were subjected to agitation to remove adhering soil and organic debris [1, 2]. One gram (1 g) of each sample was washed in sterile distilled water and surface-sterilized with ethanol (70%) for 3 minutes, hypochlorite (3%) for 3 minutes, and ethanol (70%) for 30 seconds as described by Shishido et al. [3]. The samples were finally washed five times with sterile distilled water and kept at room temperature. The sterility of the samples was assessed by plating on Luria Bertani (LB) culture media using the final rinse water from the samples. Cultured plates were incubated at 28°C, and no bacterial growth was observed on the plates after incubation.

### 2.2 DNA extraction, library preparation, and sequencing

The plant material was macerated manually with a mortar and pestle and used for DNA extraction by employing DNeasy® Plant Minikit (Qiagen, USA), following the manufacturer's protocol. The concentration and purity level of the extracted DNA were checked on 2% agarose gel and electrophoresed. The targeted bacterial V4 region of the 16S rRNA gene was amplified with forward – 515f / reverse – 806r primers as described by Caporaso et al. [4]. The PCR reactions were performed in a 30 cycle (5 cycles used on PCR products) using HotStar Taq Plus Master Mix (Qiagen, USA). The PCR amplification process was performed on ThermalCycler machine (BIO-RAD, USA, C1000 Touch^TM^), programmed at initial denaturation 94°C for 3 minutes, followed by 30 cycles of denaturation at 94°C for 30 seconds, annealing at 53°C for 40 seconds, and extension at 72°C in 1 minute, followed by a final extension at 72°C for 5 minutes and incubated at 4°C. Multiple samples were pooled together in equal proportions based on their molecular weight and DNA concentrations. Pooled samples were purified using calibrated Ampure XP beads following the instructions of the manufacturer. The purified PCR product was used to prepare the DNA library, following the DNA library preparation kit protocol. Sequencing was performed at MR DNA molecular laboratory (www.mrdnalab.com, Shallowater, TX, USA) on the Illumina MiSeq platform following standard Illumina protocol. The sequence raw dataset in FASTQ file type was demultiplexed using FASTQ Splitter 64 bit v19.07.10.

### 2.3 Sequence data analysis

Sequence read processing was done using Quantitative Insights Into Microbial Ecology (QIIME 1) 16S pipeline (version 1.9) [5] based pipeline implemented on Nephele microbial bioinformatics platform (version 1.8) (https://nephele.niaid.nih.gov/) [6]. The preprocessing steps included read pair joining using default parameters (% maximum difference of 25, and a minimum overlap of 10), removal of reads with an average Phred score of ≤ 20, removal of chimeras using VSEARCH [7], and clustering, using Open Reference Method and SILVA 99 version 132 [8]. Taxonomy assignments were done against SILVA version 132, with a default sequence similarity threshold of 0.99. The chimeric sequences such as mitochondria, chloroplast, and singleton reads were removed.

## CRediT Author Statement

**Olubukola Oluranti Babalola:** Conceptualization, Data curation, Funding acquisition, Investigation, Project administration, Resources, Supervision, and Writing-review and editing; **Bartholomew Saanu Adeleke:** Data curation, Formal analysis, Investigation, Methodology, Writing-original draft, and Writing-review and editing; **Ayansina Segun Ayangbenro:** Data curation, Formal analysis, Investigation, and Writing-review and editing.

## Declaration of Competing Interest

The authors declare that they have no known competing financial interests or personal relationships which have or could be perceived to have influenced the work reported in this article.

## Appendix Supplementary materials

Image, application 1

Image, application 2

Image, application 3

Image, application 4

Image, application 5

Image, application 6

Image, application 7

Image, application 8

Image, application 9

Image, application 10

Image, application 11

Image, application 12

Image, application 13

Image, application 14

Image, application 15

Image, application 16

Image, application 17

Image, application 18

Image, application 19

Image, application 20

Image, application 21

Image, application 22

Image, application 23

Image, application 24
