# Supplementary material for: 16S rRNA gene amplicon sequence data from sunflower endosphere bacterial community
Source: Data Brief. 2021 Nov 26;39:107636. doi: 10.1016/j.dib.2021.107636 (PMC8639433; doi:10.1016/j.dib.2021.107636)
Supplement: Supplementary file 1 [file mmc1.docx]

**Table S1:** Summary of the sequence read count

| Sample | Total read count | Minimum | Maximum | Median | Mean |
| --- | --- | --- | --- | --- | --- |
| Growing | 237,439 | 48,763 | 74,158 | 57,259 | 59,360 |
| AGR |  |  |  |  |  |
| AGS |  |  |  |  |  |
| BGR |  |  |  |  |  |
| BGS |  |  |  |  |  |
| Flowering | 331,510 | 60,480 | 108,015 | 81,508 | 82,878 |
| AFR |  |  |  |  |  |
| AFS |  |  |  |  |  |
| BFR |  |  |  |  |  |
| BFS |  |  |  |  |  |

**Key:** **Growing** (AGR –root samples from Lichtenburg, BGR−root samples from Itsoseng, AGS−stem samples from Lichtenburg, BGS−stem samples from Itsoseng); **Flowering** (AFR–root samples from Lichtenburg, BFR−root samples from Itsoseng, AFS−stem samples from Lichtenburg, BFS−stem samples from Itsoseng)
